# Ultrafast Sintering of Dense Li_7_La_3_Zr_2_O_12_ Membranes for Li Metal All‐Solid‐State Batteries

**DOI:** 10.1002/advs.202412370

**Published:** 2024-11-18

**Authors:** Faruk Okur, Huanyu Zhang, Julian F. Baumgärtner, Jaka Sivavec, Matthias Klimpel, Gregor Paul Wasser, Romain Dubey, Lars P.H. Jeurgens, Dmitry Chernyshov, Wouter van Beek, Kostiantyn V. Kravchyk, Maksym V. Kovalenko

**Affiliations:** ^1^ Laboratory of Inorganic Chemistry Department of Chemistry and Applied Biosciences ETH Zürich Zürich CH‐8093 Switzerland; ^2^ Laboratory for Thin Films and Photovoltaics Empa Swiss Federal Laboratories for Materials Science & Technology Dübendorf CH‐8600 Switzerland; ^3^ Laboratory for Joining Technologies & Corrosion Empa – Swiss Federal Laboratories for Materials Science & Technology Dübendorf CH‐8600 Switzerland; ^4^ Swiss‐Norwegian Beamlines European Synchrotron Radiation Facility Grenoble 38000 France

**Keywords:** Li metal all‐solid‐state batteries, lithium, LLZO, membranes, solid‐state electrolyte

## Abstract

Ultrafast sintering (UFS) is a compelling approach for fabricating Li_7_La_3_Zr_2_O_12_ (LLZO) solid‐state electrolytes (SSEs), paving the way for advancing and commercializing Li‐garnet solid‐state batteries. Although this method is commonly applied to the sintering of LLZO ceramics, its use for producing dense, phase‐pure LLZO SSEs has thus far been primarily limited to millimeter‐thick pellets, which are unsuitable for commercial solid‐state batteries. This study presents ultrafast sintering as a highly effective approach for fabricating self‐standing, dense, 45 µm‐thick LLZO membranes. The chemical and structural evolution of LLZO membranes during the UFS process is characterized through in situ synchrotron X‐ray diffraction and thermogravimetric analysis‐mass spectrometry, complemented by an in‐depth investigation of surface chemistry using X‐ray photoelectron spectroscopy. The membranes in Li/LLZO/Li symmetrical cell configuration exhibit a high critical current density of up to 12.5 mA cm^−2^ and maintain superior cycling stability for 250 cycles at a current density of 1 mA cm^−2^, with an areal capacity limit of 1 mAh cm^−2^. The electrochemical performance of LLZO membranes is also assessed in full cell configuration using a pyrochlore‐type iron (III) hydroxy fluoride cathode.

## Graphical abstract

In this research article, the authors report on the ultrafast sintering as a compelling methodology for fabrication of self‐standing dense LLZO membranes. The membranes exhibit in Li/LLZO/Li symmetrical cell configuration a high critical current density of up to 12.5 mA cm^−2^ and maintain superior cycling stability for 250 cycles at a current density of 1 mA cm^−2^, with an areal capacity limit of 1 mAh cm^−2^.

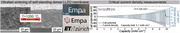

## 1 Introduction

The field of battery research is rapidly shifting toward developing Li_7_La_3_Zr_2_O_12_ (LLZO)‐based solid‐state lithium metal batteries.^[^ ^1^, ^2^, ^3^ ^]^ These systems are expected to address the growing need for safe, non‐flammable, and temperature‐tolerant energy storage solutions while offering improved energy density and extended cycle life compared to conventional liquid‐electrolyte batteries.^[^ ^4^, ^5^, ^6^, ^7^, ^8^, ^9^, ^10^, ^11^, ^12^, ^13^, ^14^, ^15^ ^]^ To date, the production of LLZO solid‐state electrolytes (SSEs) and their integration into batteries still face significant hurdles, with sintering processes particularly challenging.^[^ ^16^ ^]^ Key obstacles include maintaining the correct lithium stoichiometry for the cubic LLZO structure, achieving consistent microstructure reproducibility, and overcoming the high costs and process scalability limitations.

A new ultra‐fast sintering (UFS) method has been proposed recently to overcome the challenges associated with sintering LLZO SSEs.^[^ ^17^, ^18^, ^19^ ^]^ This technique utilizes Joule heating, enabling it to reach the sintering temperature of LLZO within seconds. Applying thermal shock promotes particle fusion, thus reducing the sintering time and lowering the sintering temperature of LLZO ceramics. This, in turn, helps to minimize lithium losses, which could otherwise result in the formation of a poorly conductive secondary La_2_Zr_2_O_7_ (LZO) phase.

The first successful application of this technique for sintering LLZO ceramics was demonstrated by Hu et al. in 2020.^[^ ^17^ ^]^ Subsequent studies from various research groups have reported the electrochemical performance of LLZO ceramics sintered using UFS in Li/LLZO/Li symmetric configurations.^[^ ^19^, ^20^, ^21^, ^22^, ^23^, ^24^, ^25^, ^26^ ^]^ Despite these advancements, the use of UFS for producing phase‐pure, fully dense, and self‐standing LLZO SSEs has only been demonstrated on millimeter‐thick LLZO pellets, which cannot be used in commercial solid‐state batteries. As highlighted by Kravchyk et al.,^[^ ^6^ ^]^ using LLZO SSEs with a thickness of less than 100 µm is essential for achieving volumetric energy densities comparable to conventional Li‐ion batteries. For example, in an all‐solid‐state battery employing LLZO and a conventional LiCoO_2_ cathode, an LLZO membrane with a thickness of 85 µm, paired with a cathode of 3.5 mAh cm^−2^, is required to achieve an energy density of 700 Wh L^−1^. To reach energy densities as high as 900 Wh L^−1^, even thinner LLZO membranes of *ca*. 40 µm are necessary.

In this study, we showcase the effectiveness of the UFS technique in producing phase‐pure LLZO SSEs as self‐standing, dense membranes. Our results demonstrate that UFS enables the fabrication of LLZO membranes with a density of up to 99% and thicknesses under 50 µm. The structural and morphological transformations during the sintering process were thoroughly examined using synchrotron X‐ray diffraction (SXRD). Additionally, the surface properties of the UF‐sintered LLZO membranes were comprehensively analyzed through X‐ray photoelectron spectroscopy (XPS) combined with argon sputtering. In a symmetrical Li/LLZO/Li cell configuration, the resulting LLZO membranes exhibited a high critical current density (CCD) of up to 12.5 mA cm^−2^. Li plating/stripping experiments demonstrated superior cycling stability of LLZO membranes over 250 h at a current density of 1 mA cm^−2^ and an areal capacity limit of 1 mAh cm^−2^.

## 2 Results and Discussion

**Figure 1:**
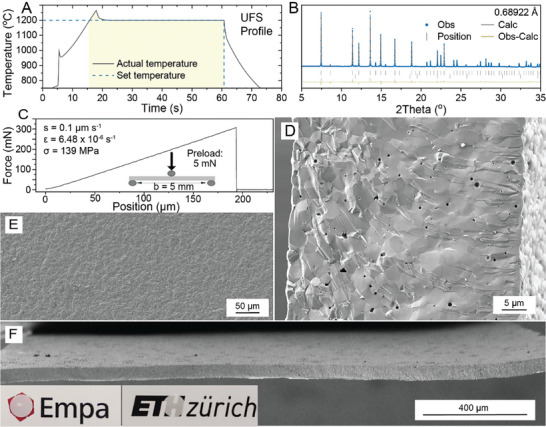
A) A typical temperature profile used for UFS of LLZO membranes. B) SXRD pattern of ultrafast‐sintered LLZO membranes. C) The force‐displacement curve of the ultrafast‐sintered LLZO membrane was measured by a three‐point bending test at a crosshead loading speed of 0.1 µm s^−1^ (inset: the configuration of the 3‐point bending setup). E) Top‐view and D,F) cross‐sectional SEM images of ultrafast‐sintered LLZO membranes. Photographs of the membranes are shown in the inset.

Figure S1 (Supporting Information) outlines the main steps in the preparation of self‐standing dense LLZO membranes, which include: 1) preparation of the slurry and 2) its tape casting on a glass substrate, 3) drying and peeling of the obtained tapes, 4) cutting of the tapes followed by 5) de‐binding and 6) ultrafast sintering. The scanning electron microscopy (SEM) images of used LLZO powder and LLZO tapes after the de‐binding step are shown in Figure S2 (Supporting Information). To determine the optimal sintering conditions of the de‐binded LLZO membranes, a series of UFS experiments were conducted with varying sintering times and temperatures (15–105 s and 1100–1300 °C, respectively; see Figure S3, Supporting Information). Our findings indicate that sintering at temperatures below 1200 °C for less than 45 s produced highly porous LLZO membranes. Conversely, temperatures above 1200 °C and sintering times exceeding 80 s resulted in over‐densification, i.e., fragile melted membranes. XRD and SEM measurements of LLZO membranes sintered at 1200 °C for 45 s confirmed (**Figure** **1**B,D–F; Figure S2, Supporting Information) that developed membranes are characterized by a high density of 5.08 g cm^−3^ (99% from the theoretical value of 5.1 g cm^−3^) and exhibited a phase‐pure cubic LLZO structure (space group $\mathrm{Ia}\bar{3}d$, with lattice parameters *a* = 12.9622(2)  Å and volume *V* = 2177.89 Å^3^, ICSD 235 896). Importantly, three‐point bending tests demonstrated the mechanical robustness of these membranes, with a breaking force of 307 mN, corresponding to a flexural strength of 139 MPa at 193.5 µm displacement (Figure 1C).

**Figure 2:**
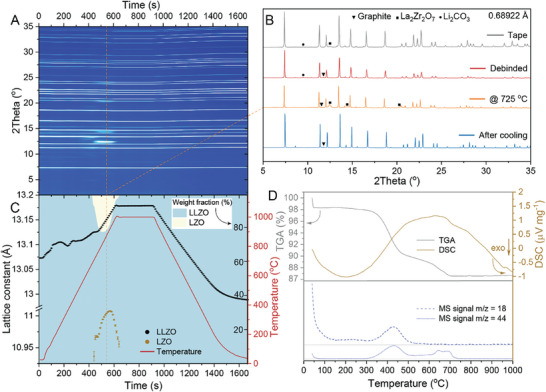
A) SXRD map of the de‐binded LLZO membrane measured during its ultrafast heat‐treatment to 1000 °C at a heating rate of 100 °C min^−1^. B) SXRD patterns of de‐binded LLZO membrane at different stages of heat treatment. The XRD pattern of the LLZO tape is shown for comparison. C) The variations of lattice constants and weight fractions of cubic LLZO and La_2_Zr_2_O_7_ phases refined during heat‐treatment of de‐binded LLZO membrane. The R‐factor of the sequential Rietveld refinement is shown in Figure S4 (Supporting Information). D) The curves of thermal gravimetric analysis, differential scanning calorimetry, and mass spectrometry measured during heat‐treatment of de‐binded LLZO membrane to 1000 °C in Ar atmosphere at heating rate of 25 °C min^−1^.

To elucidate the chemical transformations occurring during the sintering of de‐binded LLZO membranes, in situ SXRD measurements were employed to analyze the membranes during their heat‐treatment (HT) up to 1000 °C under an inert atmosphere (**Figure** **2**A–C). The heating rate was set to *ca*. 100 °C min^−1^ to simulate UFS conditions. In situ SXRD measurements were complemented by thermogravimetric analysis coupled with mass spectrometry (TGA‐MS) conducted on identically produced de‐binded LLZO membranes during their heating up to 1000 °C at a heating rate of 25 °C min^−1^ under an inert atmosphere (Figure 2D). These analyses revealed multiple chemical processes occurring during sintering.

The appearance of MS signals for H_2_O (m/z = 18) and CO_2_ (m/z = 44), along with mass losses of ca. 2 wt.%, indicates that physical desorption of H_2_O and CO_2_ occurs in the temperature range of RT‐100 °C during the heating of the de‐binded LLZO membrane. Upon further increasing the temperature to ≈150 °C, LiOH peaks were observed in the SXRD patterns, as shown in Figure 2A and Figure S5 (Supporting Information). It is hypothesized that LiOH initially formed in an amorphous state due to a chemical reaction between the pristine LLZO powder and moisture during slurry and tape preparation steps and then crystallized at 150 °C. According to previous studies,^[^ ^27^, ^28^, ^29^, ^30^ ^]^ in addition to LiOH, the reaction of LLZO with water and CO_2_ produces several other minor products, including Li_2_CO_3_, La_2_(OH)_2_(CO_3_)_2_, LZO, and protonated LLZO (h‐LLZO). Notably, the presence of Li_2_CO_3_ and LZO in the as‐de‐binded LLZO membranes prior to UFS, in contrast to phase‐pure pristine LLZO powder, was confirmed by SXRD (Figure S5, Supporting Information).

Upon further increasing the temperature to *ca*. 300 °C, a small quantity of water was released, as indicated by the MS signal. Concomitantly, the LiOH peaks begin to disappear starting from 300 °C. This release of water and the disappearance of LiOH peaks are possibly associated with the deprotonation of LLZO. As the temperature increased to *ca*. 350 °C, MS showed a significant release of H_2_O and CO_2_ between 350 and 500 °C. This release was accompanied by the appearance of La_2_O_2_CO_3_ in the SXRD patterns at 350 °C (Figure S5, Supporting Information). These changes can be assigned to the decomposition of La_2_(OH)_2_(CO_3_)_2_ into La_2_O_2_CO_3_, water, and CO_2_.^[^ ^27^ ^]^ This suggests that in addition to Li_2_CO_3_, which was detectable in the SXRD patterns of de‐binded LLZO membranes, La_2_(OH)_2_(CO_3_)_2_ amorphous product was formed due to the reaction of LLZO powder with water and CO_2_ during slurry and tape preparation steps. Considering the TGA data that demonstrate mass losses of *ca*. 7 wt.% between 350–500 °C temperature range, it can be concluded that a significant quantity of La_2_(OH)_2_(CO_3_)_2_ was present in the de‐binded LLZO membranes. From 500 °C onward, a distinct MS peak was observed at 650 °C, accompanied by the appearance of the La_2_O_3_ phase ≈655 °C (Figure S5, Supporting Information). These observations likely correspond to the decomposition of La_2_O_2_CO_3_ into La_2_O_3_ and CO_2_ at *ca*. 650 °C.^[^ ^27^ ^]^

Upon further heating the LLZO membrane, the Li_2_CO_3_ reflections diminished and eventually disappeared at 750 °C. This change is associated with the melting and decomposition of Li_2_CO_3_, resulting in the formation of amorphous Li_2_O and CO_2_ gas, as evidenced by MS spectra. Following this, there was a rapid increase and subsequent decrease in the intensity of LZO reflections within the narrow temperature range of 625–770 °C, accompanied by the appearance of La_2_O_3_ secondary phases. These phenomena can be explained by the significant increase in the partial pressure of CO_2_ within the contracting pores of the densifying LLZO membrane during the thermal decomposition of Li_2_CO_3_. This increase shifts the equilibrium to favor the reaction of LLZO with CO_2_, triggering the formation of molten Li_2_CO_3_ and nanostructured LZO, as indicated by the broadening of the LZO reflections. However, this process is transient, lasting only ≈30 s, after which the equilibrium shifts back to favor the decomposition of Li_2_CO_3_ into Li_2_O and CO_2_. Subsequently, Li_2_O reacts with the nanostructured LZO, leading to the reformation of the cubic LLZO phase. The complex solid‐state reactions and phase transitions also resulted in changes of LLZO lattice constant (Figure 2C), which at the end of the in situ heat‐treatment reached the theoretical value for the cubic‐phase LLZO (*a* = 12.9622(2)  Å).^[^ ^31^ ^]^

**Figure 3:**
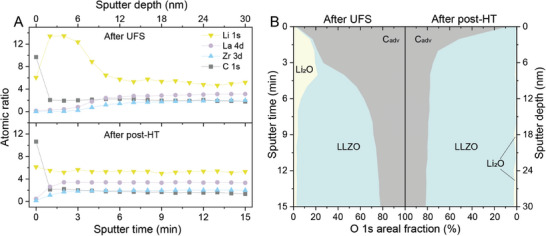
A) Calculated atomic ratio contributions based on Li 1s, La 4d, Zr 3d, and C 1s peaks of UF‐sintered LLZO membranes without and with additional heat treatment. Atomic ratio contributions are shown as a function of sputter time ranging from 0 to 15 min, normalized to the contribution of Zr as 2, consistent with LLZO stoichiometry. B) Corresponding O 1s areal fraction of LLZO, Li_2_O, and C_adv_ of UF‐sintered LLZO membranes without and with additional heat‐treatment steps. XPS surveys of the LLZO membrane are shown in Figure S6 (Supporting Information).

To ascertain the surface chemical composition of LLZO membranes following UFS, an in‐depth examination was undertaken utilizing XPS in conjunction with Ar‐ion sputter‐depth profiling (**Figure** **3**; Figures S6–S8, Supporting Information). The sputtering (by rastering a focused 1 keV Ar^+^ beam) was conducted in consecutive 1 min intervals, with each step corresponding to a sputter depth of ≈2 nm. It is noteworthy that during the transfer of the LLZO membranes to the XPS instrument, they were not exposed to air, as the XPS system is directly connected to an Ar‐purified glovebox.

The dominant C 1s peak at 284.8 eV and two minor C peaks at 286.1, and 289.1 eV, as well as O 1s peaks at 532.7, and 531.5 eV, can be attributed to C─C, C═O, O═C─O, and O═C, O═C─O chemical bonding states (see Figures S7 and S8, Supporting Information). This indicates that the surface of the UF‐sintered LLZO membrane is mainly composed of adventitious carbon (C_adv_) and its commonly associated surface species. Acknowledging the fact that the samples were not exposed to air between their preparation and subsequent XPS analysis, it must be concluded that the relatively strong C species originate from the interaction of the LLZO surface with volatile organics present in the synthesis glovebox. This contamination is practically unavoidable and has been widely reported in studies focused on XPS analysis of LLZO surface.^[^ ^32^, ^33^, ^34^, ^35^, ^36^ ^]^ The reconstructed XPS peaks at 109.0–99.0 eV (La 4d), 181.2–183.6 eV (Zr 3d), and 529.5 eV (O 1s) can be unambiguously assigned to LLZO, while the peak at 528.6 eV (O 1s) can be assigned to Li_2_O (Li─O bonds). Figure 3A shows the atomic ratios based on Li 1s, La 4d, Zr 3d, and C 1s, normalized to a Zr contribution of 2, consistent with LLZO stoichiometry. The Li and La ratios are also in agreement with the expected LLZO stoichiometry under deeper probing depths. To assess the relative chemical composition of the LLZO surface as a function of sputter depth, the areal intensities of the individual O 1s peaks at 529.5 eV (LLZO), 528.6 eV (Li_2_O), and 532.7, and 531.5 eV (C_adv_) were normalized to the total intensity of the O 1s peak envelope, further designated as the relative O 1s fraction. Accordingly, the relative O 1s fraction of each species (i.e., LLZO, Li_2_O, or C_adv_) can be traced as a function of the sputter depth up to a sputter depth of ≈30 nm (see Supporting Information for details). The results of this analysis are outlined in Figure 3B, presented as a 2D depth‐resolved chemical composition map.

According to the findings shown in Figure 3A,B, the surface composition of LLZO membranes predominantly consists of Li_2_O and C_adv_ up to a sputter depth of ca. 6 nm. Beyond this sputter depth, the surface primarily comprises the LLZO structure. The presence of Li_2_O on the outer surface of UF‐sintered LLZO membranes can be attributed to an incomplete solid‐state reaction between Li_2_O and LZO during ultrafast sintering. As discussed above, the formation of LZO and Li_2_CO_3_ arises from the reaction of pristine LLZO powder with moisture and CO_2_ during the slurry and tape‐casting preparation procedures. Subsequently, as confirmed by in situ SXRD and TGA‐MS data, Li_2_CO_3_ melts and decomposes to Li_2_O at ≈750 °C.

To eliminate the Li₂O contamination layer from the surface of UF‐sintered LLZO membranes, the latter were subjected to a post‐heat‐treatment (post‐HT) at 900 °C for 10 min in an Ar‐filled glovebox, followed by XPS surface characterization. The results are presented in Figure 3B, which displays a 2D map of chemical composition versus sputter depth. It is noteworthy that a comparison of XPS data obtained on UF‐sintered LLZO membranes without (Figure S7, Supporting Information) and with (Figure S8, Supporting Information) post‐HT demonstrates that the post‐HT effectively decreases the amount of Li₂O present on the surface of LLZO membranes by completion of the solid‐state reaction between Li_2_O and LZO to form LLZO.

Following the surface cleaning step, the LLZO membranes were characterized by electrochemical impedance spectroscopy (EIS) using an Au/LLZO/Au symmetrical cell configuration (Figure S9, Supporting Information). The estimated Li‐ion conductivity of the fabricated membranes was 0.55 mS cm^−1^ at RT, consistent with previously reported values for LLZO pellets.^[^ ^37^ ^]^ The activation energy was determined to be ca. 0.33 eV.

To evaluate the performance of the prepared LLZO membranes with respect to electrochemical Li plating and stripping, they were isostatically pressed between two Li discs at *ca*. 71 MPa to form a Li/LLZO/Li symmetric cell. As followed from EIS measurements of prepared Li/LLZO/Li symmetrical cells, the latter were characterized by low interfacial resistance of 45.3 Ω cm^2^ (Figure S10, Supporting Information), comparable with state‐of‐the‐art values measured on Li/LLZO/Li symmetrical cells based on LLZO pellets.^[^ ^38^ ^]^ Notably, EIS measurements also confirmed a significant decrease in the interfacial resistance measured on Li/LLZO/Li symmetrical cells based on post‐HT LLZO membranes compared to the ones without the post‐HT step (Figure S10, Supporting Information).

**Figure 4:**
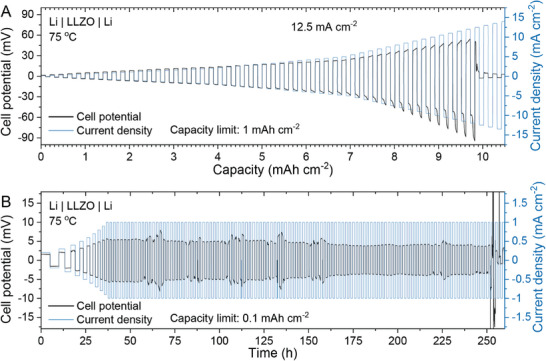
Electrochemical performance of Li/LLZO/Li symmetric cells at 75 °C. A) Voltage profile of Li/LLZO/Li symmetrical cell measured at different current densities from 0.1 to 12.5 mA cm^−2^ with an areal capacity limitation of 0.1 mAh cm^−2^ per half cycle (CCD measurements). B) Voltage profile of Li/LLZO/Li symmetrical cell, measured at a current density of 1 mA cm^−2^ with an areal capacity limitation of 1 mAh cm^−2^ per half cycle. Enlarged voltage profile regions are shown in Figure S11 (Supporting Information).

The CCD of LLZO membranes was determined through a galvanostatic cycling experiment of Li/LLZO/Li symmetrical cell across various current densities at 75 °C. The current density was increased stepwise, starting from 0.1 mA cm^−2^, with each half cycle transferring 0.1 mAh cm^−2^ of Li. **Figure** **4**A illustrates that symmetrical cells exhibited a high CCD of up to 12.5 mA cm^−2^, with minimal polarization observed at current densities below 3 mA cm^−2^. Moreover, additional galvanostatic cycling experiments conducted on the Li/LLZO/Li symmetrical cells at 1 mA cm^−2^ current rate and with an areal capacity limit of 1 mAh cm^−2^ per half cycle at 75 °C, demonstrated cycling stability of LLZO membranes over 250 h (Figure 4B). Throughout cycling, the cells displayed minimal voltage polarization of ca. 5 mV. Another Li/LLZO/Li symmetrical cell tested at a higher current rate of 1.5 mA cm^−2^ exhibited stable cycling over 40 cycles. Details and post‐mortem analysis using focused ion beam (FIB) SEM are provided in Figure S12 (Supporting Information).

**Figure 5:**
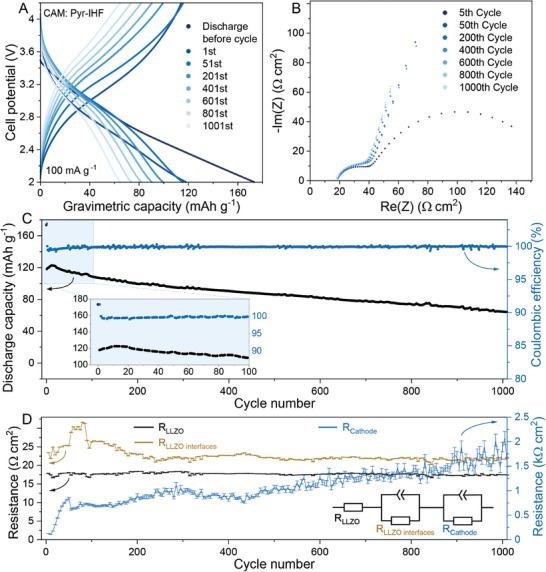
Electrochemical performance of the Li/LLZO/Pyr‐IHF full cell. The mass loading of cathode active material (CAM) was ca. 0.41 mg cm^−2^. A) Galvanostatic charge–discharge voltage profiles, C) capacity retention, and (C) coulombic efficiency of Li/LLZO/Pyr‐IHF full cell measured at a current density of 100 mA g^−1^ between 2 and 4.2 V versus Li^+^/Li. B) Impedance spectra of Li/LLZO/Pyr‐IHF full cell measured in the charge state after different number of charge–discharge cycles. D) The change of resistances for LLZO membrane (R_LLZO_), Li/LLZO, and LLZO/ionic liquid interfaces (LLZO_interfaces_) and Pyr‐IHF cathode (R_cathode_) as a function of cycle number refined from the fitting of corresponding impedance spectra of Li/LLZO/Pyr‐IHF full cell measured every 5th cycle. The equivalent circuit is shown in the inset.

Next, we probed the electrochemical performance of the LLZO membranes in a hybrid‐type full cell configuration (**Figure** **5**). The development of LLZO‐based all‐solid‐state cathodes is still in its infancy due to the chemical incompatibility of LLZO with cathode active materials at the temperatures required for LLZO co‐sintering (1050–1250 °C).^[^ ^39^ ^]^ Consequently, we used a cathode comprising an ionic liquid (IL) electrolyte to demonstrate the electrochemical performance of membranes in full‐cell configuration. As a cathode active material, pyrochlore‐type iron hydroxy fluoride (Pyr‐IHF) was employed because of its low cost, high theoretical capacity of 237 mAh g^−1^ for the one‐electron reaction, and relatively high average discharge voltage of 3 V versus Li^+^/Li.^[^ ^40^, ^41^ ^]^ Unlike conventional cathode materials, Pyr‐IHF is synthesized in its delithiated form, necessitating its pairing with a lithium metal anode. This full cell configuration can potentially achieve an energy density of up to 669 Wh kg^−1^ (theoretical value), comparable to those based on LiFePO_4_ (578 Wh kg^−1^), LiNi_1/3_Mn_1/3_Co_1/3_O_2_ (610 Wh kg^−1^) and LiCoO_2_ (546 Wh kg^−1^) cathodes.^[^ ^42^ ^]^

The full cell was composed of Li metal discs, LLZO membrane, and cathode, which was impregnated with IL electrolyte (see Supporting Information for details). In short, the cathode was prepared by ball‐milling of Pyr‐IHF with carbon black (CB) and polyvinylidene difluoride (PVDF) binder in *N*‐methylpyrrolidone (NMP) solvent. The prepared slurry was tape cast onto a carbon‐coated Al‐foil current collector, followed by drying in air and then in a vacuum at 80 °C. Li metal was isostatically pressed on one side of the LLZO membrane as described above, and the IL‐soaked Pyr‐IHF cathode was stacked on the opposite side.

Figure 5A shows the voltage profile of a full cell containing Pyr‐IHF cathodes measured between 2 and 4.2 V versus Li^+^/Li at a current density of 100 mA g^−1^ (ca. 0.4C) at RT. The initial discharge and charge capacities for the first cycle were 173 and 114 mA h g^−1^, corresponding to an initial Coulombic efficiency of 151.7%. The irreversible capacity loss during the first cycle is solely attributed to Pyr‐IHF and has been comprehensively studied in previous work on liquid‐based systems.^[^ ^41^, ^43^ ^]^ From the second cycle onward, the Coulombic efficiency improved significantly to above 99.5% for subsequent cycles. The results demonstrate a high capacity retention of 80% after 300 cycles, and a total cycle number of 1000 cycles, indicating the high cyclic stability of the designed full cells. The resistances of the full cell were also investigated by EIS measurements after every 5 cycles on the charged state (Figure 5B,D and Figure S13). Three separate contributions to the resistance were fitted in the equivalent circuit, namely, LLZO_bulk_, LLZO_interfaces_ (Li‐LLZO and IL‐LLZO), and Pyr‐IHF total resistance (including all the components and interfaces in the cathode). Notably, the resistance of LLZO_bulk_ and LLZO_interfaces_ remained stable upon 1000 cycles, except the LLZO interface stabilization cycles for the first 100 cycles. The resistance associated with Pyr‐IHF increased during cycling, presumably causing the capacity decay during cycling. Notably, the voltage profiles and cycling stability of the full cells were similar to those of liquid‐state full cells without the LLZO membrane,^[^ ^41^ ^]^ underscoring the potential applicability of the developed LLZO membranes for use in hybrid full cell configurations with IL electrolytes. In addition to the Pyr‐IHF based full cells with IL electrolyte, we also prepared all‐solid‐state batteries based on LiNi_0.8_Mn_0.1_Co_0.1_O_2_ (MNC811, loading 7 mg cm^−2^) cathode active material and Li_6_PS_5_Cl (LPSCl) catholyte (see Supporting Information for details). The prepared Li/LLZO/NMC811‐LPSCl full cells were measured at a current density of 20 mA g^−1^ (0.1C) between 3 and 4.3 V versus Li^+^/Li and showed a high initial capacity of 1.2 mAh cm^−2^ (Figure S14, Supporting Information). Notably, the gravimetric and volumetric energy densities of the prepared cells were estimated to be *ca*. 258 Wh kg^−1^ and 888 Wh L^−1^, respectively (Table S1, Supporting Information).

## 3 Conclusion

In conclusion, we present UFS as a compelling method for fabricating self‐standing, dense, 45 µm‐thick LLZO membranes. Through a series of UFS experiments with varying sintering times (15–105 s) and temperatures (1100–1300 °C), we found that sintering at 1200 °C for 45 s yields mechanically stable, phase‐pure LLZO membranes with a density of ca. 5.08 g cm^−3^ (ca. 99% from the theoretical value of 5.1 g cm^−3^). These membranes exhibited exceptional mechanical strength, with a breaking force of 307 mN.

Our study also addresses a critical challenge in UFS of LLZO membranes: the formation of 8–12 nm thick Li_2_O contamination layer on the membrane surface, as revealed by the comprehensive characterization of the LLZO surface by XPS coupled with argon sputtering. Using in situ SXRD in combination with TGA‐MS measurements, we determined that this contamination results from an incomplete solid‐state reaction between Li_2_O and LZO during UFS. The presence of LZO in de‐binded LLZO membranes is attributed to the reaction of LLZO powder with H_2_O and CO_2_ during its storage and handling in air, forming LZO and Li_2_CO_3_. The latter decomposes to Li_2_O and CO_2_ during sintering. Further investigations have shown that a post‐HT of UF‐sintered LLZO membranes at 900 °C effectively enables the completion of the Li_2_O/LZO reaction, thereby mitigating Li_2_O surface contamination. After thermal surface cleaning of the LLZO membranes at 900 °C for 10 min in an argon atmosphere, we thoroughly evaluated their electrochemical properties. EIS measurements in a Au/LLZO/Au symmetric cell configuration revealed that the membranes possess high Li‐ion conductivity at the level of 0.55 mS cm^−1^ at room temperature. The membranes also exhibited a high CCD of 12.5 mA cm^−2^ and a cycling stability of more than 250 h at 1 mA cm^−2^ with an areal capacity limit of 1 mAh cm^−2^ at 75 °C. The electrochemical performance of the dense LLZO membranes was also evaluated in a full‐cell configuration with a Pyr‐IHF cathode. The Li/LLZO/Pyr‐IHF full cells demonstrated superior electrochemical cycling stability, maintaining a high gravimetric capacity of 94 mAh g^−1^ at a current density of 100 mA g^−1^ (0.4C) after 300 cycles.

## Conflict of Interest

The authors declare no conflict of interest.

## Supporting information



Supporting Information

## Data Availability

The data that support the findings of this study are available from the corresponding author upon reasonable request.
