# Supplementary material for: Ultrafast Sintering of Dense Li7La3Zr2O12 Membranes for Li Metal All‐Solid‐State Batteries
Source: Adv Sci (Weinh). 2024 Nov 18;12(2):2412370. doi: 10.1002/advs.202412370 (PMC11727369; doi:10.1002/advs.202412370)
Supplement: Supplementary file 1 — Supporting Information [file ADVS-12-2412370-s001.docx]

Supporting Information

Ultrafast Sintering of Dense Li_7_La_3_Zr_2_O_12_ Membranes for Li Metal All-Solid-State Batteries

Faruk Okur, Huanyu Zhang, Julian F. Baumgärtner, Jaka Sivavec, Matthias Klimpel, Gregor Paul Wasser, Romain Dubey, Lars P.H. Jeurgens, Dmitry Chernyshov, Wouter van Beek, Kostiantyn V. Kravchyk,^*^ Maksym V. Kovalenko^*^

**Chemicals**

Al-LLZO (Ampcera, 500 nm nanopowder, Al-LLZO), isopropanol (Emsure), ethanol (Sigma-Aldrich), 1-propanol (99.5%, AcroSeal), isopropanol (Emsure), surfactant solution (Malialim, NOF Corporation), plasticizer solution (G-260, Sekisui Chemical) and polyvinyl butyral binder (PVB - BL-7Z, Sekisui Chemical, Mw 3.91×10^4^), FeF_3_(H_2_O)_2_ ⋅ H_2_O (98%, abcr chemicals), EtOH (absolute for analysis, 99.8%, EMSURE, ACS), FeF_3_(H_2_O)_2_ ⋅ H_2_O (abcr chemicals), Li metal rod (Sigma Aldrich, 99.9%) Lithium bis(fluorosulfonyl)imide (LiFSI, 99%, Solvionic), 1-butyl-1-methylpyrrolidinium bis(fluoromethanesulfonyl)imide (PYR14FSI, 99%, Iolitec), polyvinylidene difluoride (PVDF, average Mw ~534 g mol^-1^, Sigma Aldrich), N-methylpyrrolidone (NMP, 99%, Sigma Aldrich), carbon black (CB, Super P Li, Imerys), LiNbO_3_ coated single crystal LiNi_0.8_Mn_0.1_Co_0.1_O_2_ (NMC811, KRI Inc.), Li_6_PS_5_Cl (LPSCl, D50 = 3 µm, Wellcos), vapor grown carbon fiber (VGCF, 99.99%, fiber diameter 150 nm, fiber length 4 µm, Resonac), hydrogenated butadiene acrylonitrile ter-polymer (HNBR, Therban® LT 1707, Arlanexo), Toluene (>99.5%, Roth), isobutyl isobutyrate (IBIB, > 98%, Sigma Aldrich).

**Preparation of LLZO slurry and tape-casting process**

In the typical slurry preparation procedure, 0.4 g of G-260 plasticizer solution was mixed with 0.43 g of Malialim surfactant solution and 4.3 mL of a solvent mixture (isopropanol: ethanol: 1-propanol = 5:87:8 vol%) in a glass vial. The resulting solution (*ca*. 5.1 mL) was then mixed with 3 g of LLZO powder in a ball-milling jar, initially stirred with a spatula and subsequently ball-milled at 165 rpm for 18 hours. Afterward, 2 mL of binder solution (PVB: isopropanol = 30:70 wt.‑%) was added to the LLZO suspension, thoroughly mixed with a spatula, and further ball-milled at 200 rpm for 2 hours. The resulting LLZO slurry was tape-casted onto a glass substrate at a speed of 1 mm s^−1^ using a doctor blade with an opening gap of 400 µm. The tape-casted layer was air-dried for 30 minutes, peeled off from the glass substrate, and laser-cut into 10 mm diameter discs for subsequent de-binding and sintering steps. The solid content composition of the prepared tape-cast disks was as follows: 68.7 wt.‑% LLZO, 8.9 wt.‑% G-260 plasticizer, 9.7 wt.‑% Malialim surfactant, and 12.6 wt.‑% polyvinyl butyral binder.

**De-binding of LLZO tapes**

The tape-cast LLZO discs were placed between two flat, micro-porous alumina plates and then heat-treated with the following temperature profile in a muffle furnace under an O_2_ flow: 150 °C for 2 hours, 400 °C for 2 hours and 600 °C for 2 hours.

**Ultrafast sintering (UFS) of de-binded LLZO membranes**

The sintering of the de-binded LLZO membranes was conducted using a custom-made setup (**Figure S1**) under an argon atmosphere in a glovebox. The UFS setup was powered by an alternating current/direct current (AC/DC) source (Aim-TTi CPX400DP Dual 420 W PowerFlex DC Power Supply). The sintering temperature was monitored with an IR camera (MAURER Pyrometer KTRD 4085-1). The de-binded LLZO membranes were positioned between graphite foils and two boron nitride plates. This assembly was placed within the slit of the carbon felt in the UFS setup. The detailed configuration of the UFS setup is shown in our previous study.^[19]^ The UFS process was carried out at 1200 °C for 45 seconds. A typical experimental sintering profile is shown in **Figure 1a**. The selected sintering temperature of 1200 °C was based on ex-situ SEM experiments of LLZO membranes performed in our previous work,^[1]^ which indicated that substantial densification of the LLZO membranes begins around 1100 °C.

**Surface cleaning of UF-sintered LLZO membranes**

To eliminate Li_2_O residues on the LLZO surface after UFS, the sintered LLZO membranes were subjected to a post-HT at 900 °C for 10 minutes in a muffle furnace (Nabertherm) inside an argon-filled glovebox.

**Synthesis of Pyr-IHF**

The synthesis of Pyr-IHF has been described previously.^[2]^ FeF_3_(H_2_O)_2_ ⋅ H_2_O (500 mg) was partially dissolved in EtOH (150 mL) for 6 hours at room temperature, forming an iron hydroxy fluoride ethanolic solution. The suspension was centrifuged (10000 rpm, 5 min) and the clear yellow solution was decanted and filtered through a PTFE syringe filter (0.45 μm). Subsequently, 6 mL of distilled water was added to the solution and allowed to precipitate for 24 h. The suspension was then centrifuged (10000 rpm, 5 min), and the residual beige powder of Pyr-IHF was washed with EtOH. Pyr-IHF was then dried under vacuum at 80 °C.

**Heat Treatment of Pyr-IHF**

The heat-treatment of Pyr-IHF has been described previously.^[2]^ As-synthesized Pyr-IHF (*ca*. 100 mg) was heated to 280 °C for 30 min in a tube furnace (Carbolite Gero) in air at a heating rate of 10 °C min^−1^, followed by a natural cooling rate by switching off the oven.

**Materials Characterization**

**Thermogravimetric Analysis/Mass Spectrometry (TGA-MS)**

TGA-MS measurements of raw LLZO powder, and de-binded LLZO membranes were conducted over a temperature range of 50 – 1100 °C with a heating rate of 25 °C min^−1^ in an argon flow of 20 mL min^−1^. The heating rate was limited by the instrument. These measurements were performed using a Netzsch Simultaneous Thermal Analyzer (STA 449 F5 Jupiter) combined with a Netzsch Quadrupole Mass Spectrometer (403 D Aëolos Quadro). Alumina crucibles were used as sample holders. The balance resolution of the TGA was 0.1 µg.

**Scanning Electron Microscopy (SEM)**

SEM imaging of the LLZO powder, surface and cross-section of the dense LLZO membranes were performed using a Zeiss Gemini 460 with a secondary electron detector at 5-10 kV acceleration voltage and 100 pA current. Additional SEM images, shown in **Figure S3**, were obtained with a Hitachi TM3030Plus Tabletop microscope, operating at an acceleration voltage of 10 kV.

**Focused Ion Beam Scanning Electron Microscopy (FIB-SEM)**

Focused ion beam scanning electron microscopy (FIB-SEM) cross-section images were obtained using a Thermo Fisher Scientific Helios 5 Laser Hydra System. The sample was loaded with minimal air exposure into the chamber. To avoid any shadow effect, a 600-µm wide cross-section was opened using the femto-second laser at a wavelength of 1030 nm, a pulse rate of 60 kHz and a power of 4 mJ. The obtained surface was polished using the Argon plasma at 30 kV and a current of up to 0.93 µA. The images were recorded using an Everhart–Thornley detector with 2 kV acceleration voltage and 1.6 nA current with tilt correction enabled.

**Measurement of mechanical properties**.

A 3-point bending test of the fabricated LLZO membranes was conducted using a Tinius Olsen 1ST electromechanical testing machine. The test applied a crosshead speed (*s*) of 0.1 µm s^−1^ and a strain rate ($\varepsilon$) of 1.56 × 10^−5^ s^−1^ to the sample. Flexural strength ($\sigma_{f}$) and strain rate ($\varepsilon$) were calculated using the following formulas:

$\sigma_{f}=\frac{3000FL}{{2bd}^{2}}$ (MPa) (1),

$\varepsilon=\frac{6ds}{L^{2}}$ (s^-1^) (2),

where $b$ is the diameter of the LLZO membrane (8 mm, circular-shaped), $d$ is the thickness of the LLZO membrane (45 μm), $F$ is the breaking force (mN), and $L$ is the support span (5 mm).

***In-situ* Synchrotron X-Ray Diffraction Measurement (SXRD)**

*In-situ* SXRD experiments were conducted at the Swiss-Norwegian Beamline (SNBL), BM01, at the European Synchrotron Radiation Facility (ESRF) using the PILATUS@SNBL diffractometer (λ = 0.68966 Å) in high-intensity beam mode (∼200 mA). A custom-made furnace was used for the experiments, which were carried out under a nitrogen atmosphere.^[3]^ The furnace was heated gradually at a ramp rate of 100 °C per minute up to 1000 °C. Data acquisition time was 10 seconds per pattern. The 2D diffraction data from the Pilatus 2M detector were processed using the SNBL Toolbox and BUBBLE software. The analysis of SXRD peaks was performed using the Modulation-Enhanced Diffraction Viewer and Editor (Medved) software, developed by SNBL.^[4]^

Sequential Rietveld refinement of *in-situ* heat-treatment was performed with the *WinPLOTR* software. Instrumental parameters were determined with a LaB_6_ NIST standard. Three phases are included to refine spectra at each temperatures: cubic phase LLZO (Ia$\bar{3}$d, ICSD code 235896), La_2_Zr_2_O_7_ (Fd$\bar{3}$m, ICSD code 26851), and graphite (P6_3_/mmc, ICSD code 76767). The background was modeled with polynomial function based on 16 background data points. During sequential Rietveld refinement, background, lattice parameters (a, b, c), phase fractions (scaling factors), profile shape parameters and overall isotropic displacement parameters were varied for all three phases. The atoms positions, individual isotropic displacement parameters, and occupation numbers were kept as same as cif files from ICSD database.

**XPS analysis**

XPS measurements were conducted using a PHI Quantes spectrometer (ULVAC-PHI) with a focused monochromatic Al-Kα source (*h*ν = 1486.6 eV). The spectrometer was directly connected to an Ar glovebox (with an oxygen and water purifier system, O_2_ < 0.1 ppm H_2_O < 0.5 ppm), allowing for the *in-situ* transfer of LLZO membranes from the Ar synthesis glovebox to the XPS glovebox without intermediate air exposure. The linearity of the energy scale of the hemispherical analyzer was calibrated according to ISO 15472 by referencing the Au 4f^7^/_2_, Ag 3d^5^/_2_ and Cu 2p^3^/_2_ main peaks to the recommended binding energy (BE) positions of 83.96 eV, 368.21 eV, and 932.62 eV, respectively (as measured in situ for the sputter-cleaned, high-purity metal references). To maintain charge neutrality during measurements, a dual beam charge neutralization system was used, employing low-energy electron and argon-ion beams with a 1 V bias and a 20 µA current. Compositional sputter-depth profiles were obtained through alternating cycles of XPS analysis (Al-Kα at 51 W; beam diameter approximately 200 μm) and sputtering with a focused 1 keV argon beam, rastering an area of 2×2 mm^2^. After each successive sputter step, the Li 1s, La 4d, Zr 3d, C 1s, and O 1s regions were recorded with a step size of 0.2, 0.1, 0.2, 0.2, and 0.2 eV respectively and with a pass energy of 112 eV. The etch rate was calibrated to 2 nm min^−1^ using a 100 nm Ta_2_O_5_/Ta reference sample. XPS survey spectra covering the binding energy range of 0 eV to 1200 eV were recorded with a step size of 0.8 eV at a constant pass energy of 224 eV using the Al-Kα source (power 51 W; beam diameter approximately 200 μm). The probing depths for the La 4d, Zr 3d, O 1s, C 1s, and Li 1s photoelectron lines, recorded from LLZO with Al-Kα X-ray radiation are: O 1s (4.09 nm), C 1s (4.92 nm), Zr 3d (5.24 nm), La 4d (5.50 nm), and Li 1s (5.65 nm).

To correct for differential charging of the insulating LLZO surfaces, the photoelectron spectra were charge-corrected by adjusting the adventitious C 1s peak (C-C bond) to the reference value of 284.8 eV. Spectral reconstruction was performed using CasaXPS software, employing linear-least squares fitting of background-corrected spectra with symmetric, mixed Gaussian–Lorentzian line shape functions. The Gaussian fraction for each peak component was fixed at 0.5. The full width at half maximum (FWHM) and relative BE position of each fitted peak component were kept constant across the sputter depth. The atomic ratio of different elements were first estimated by CasaXPS with the relative sensitive factors of Li 1s (0.0568), La 4d (7.25459), Zr 3d (8.0256), and C 1s (1.00). The atomic ratio were afterwards normalized to the contribution of Zr as 2, consistent with LLZO stoichiometry. The areal fraction of each O 1s peak was calculated from its integrated peak area at each sputter cycle. The binding energy values in this study have a general deviation of ±0.1 to 0.2 eV due to instrumental error.

**Electrochemical Impedance Spectroscopy (EIS) measurements**

The Li-ion conductivity of dense LLZO membranes was measured in the temperature range of -30 °C to 100 °C using EIS. These measurements were conducted in an Au/LLZO/Au symmetrical cell configuration within an ITS-e Temperature Chamber (Biologic), equipped with a CESH-e sample holder (Biologic). Au electrodes, approximately 50 nm thick, were thermally evaporated onto ultrafast-sintered LLZO membranes using a Covap thermal evaporator (Angstrom). Before Au evaporation, the LLZO membranes were subjected to a post-HT at 900 °C for 10 minutes in an argon-filled glovebox using a muffle furnace. This step was performed to eliminate Li_2_O contamination from the surface, as confirmed by XPS analysis. EIS measurements were carried out over a frequency range of 35 MHz to 10 Hz with an amplitude of 10 mV, using an MTZ-35 impedance analyzer (Biologic). The ionic conductivities (*σ*, S cm^-1^) were calculated based on the total resistance (*R*, Ω) values of the dense LLZO membranes, considering their thickness (*l* = 45 µm) and the surface area of the Li electrodes (*S* = 0.12566 cm^2^), with following equation:

$\sigma=\frac{l}{R\times S}$ (3),

The activation energy calculation in **Figure S9a** was calculated by the Arrhenius equation:

$\sigma T=Ae^{\frac{{-E}_{a}}{k_{B}T}}$ (4),

where *A* is the pre-exponential factor, *E_a_* (eV) is the activation energy for ionic conduction, *T* (K) is the temperature, and *k_B_* (eV K^-1^) is the Boltzmann constant.

**Preparation of Li/LLZO/Li symmetrical cells**

Li/LLZO/Li symmetrical cells were prepared by cold isostatic pressing of Li foil discs (71 MPa for 3 minutes) on both sides of dense LLZO membranes in an inert environment using a PW 100 EH cold isostatic press (P/O/Weber). The Li foil was prepared by cutting a Li metal rod into small pieces, rolling them on a stainless steel substrate to a thickness of *ca*. 100 µm, and then cutting 4 mm discs from the resulting foil.

Electrochemical measurements of the symmetrical cells were conducted at 75 °C in a sealed vacuum furnace integrated within an argon-filled glovebox, connected to a BioLogic VMP-300 multichannel workstation. EIS measurements were performed in the frequency range of 10 Hz to 7 MHz with an amplitude of 10 mV using the BioLogic VMP-300 multichannel workstation.

**Pyr-IHF cathode preparation**

The synthesis of Pyr-IHF has been described previously.^[41]^ A slurry was prepared by mixing heat-treated Pyr-IHF (70 mg, 50 wt.‑%), CB (56 mg, 40 wt.‑%) and a solution of 0.833 wt‑% pVdF binder in NMP (14 mg, 10 wt.‑% pVdF; 1680 mg, 1200 wt.‑% NMP), followed by ball-milling of the obtained suspension in a ZrO_2_ beaker (12 mL) with ZrO_2_ balls (20 g, 5 mm Ø) for 1 h at 300 rpm using a planetary ball-mill (Fritsch, Pulverisette 7). The resulting slurry was then immediately tape‑casted onto carbon‑coated Al foil with a doctor blade (the gab = 150 μm) at a speed of 1 mm s^‑1^. The tape‑casted Al foil was dried under air at 125 °C and then dried under vacuum at 80 °C overnight. The tape was then transferred into an Ar-filled GB without contact to air, and 4 mm Ø disks were punched out of the foil and weighed inside a GB. The mass loading of Pyr-IHF cathode active material was 0.50 mg cm^-2^.

**NMC811-LPSCl Cathode Preparation**

A 5 wt% HNBR binder stock solution was prepared by dissolving 8.76 g of HNBR in 200 mL of toluene and stirring the mixture for 24 hours. Pre-purchased powders of LNO@NMC811, LPSCl, and VGCF were weighed, transferred into a 25 mL glass jar and mixed with a spatula. The HNBR-toluene solution, along with pure toluene and IBIB, was then added to achieve the desired binder and solid material content in the slurry. The target weight ratio of LNO@NMC811:LPSCl:VGCF was 67.5:29.0:3.0:0.5, using a 50:50 vol% mixture of toluene and IBIB as the solvent. The mixture was homogenized with an IKA ULTRA TURRAX T18 homogenizer, first at 6000 rpm for 3 minutes, followed by an additional 3 minutes at 10000 rpm. The resulting slurry was tape-cast onto carbon-coated Al foil using a doctor blade set to a 200 µm gap and left to dry overnight in an Ar atmosphere.

**Full Cell preparation**

To prepare the Li/LLZO/Pyr-IHF hybrid full cells, a 4 mm lithium disc was isotatically pressed on one side of the LLZO membrane as described above. 10 µl of 1 M LiFSI in PYR_14_FSI ionic liquid was impregnated into the prepared Pyr-IHF cathode under Ar at 75 °C overnight to ensure good wetting of the cathode.^[2]^ A cellulose separator and the ionic liquid impregnated cathode were stacked on the opposite side of the LLZO membrane to construct the full cell. Galvanostatic cycling of the full cells was conducted in a voltage range between 2 – 4.2 V *vs.* Li^+^/Li at RT in an argon-filled glovebox, connected to a BioLogic VMP-300 multichannel workstation. EIS measurements were performed after every five cycle at the charged stage. The frequency range of 100 Hz to 1 MHz with an amplitude of 10 mV using the BioLogic VMP-300 multichannel workstation.

To assemble the Li/LLZO/NMC811-LPSCl all-solid-state batteries, 4 mm diameter cathode disks were punched from the NMC811-LPSCl cathode. A poly(siloxane) polymer electrolyte (PSPE) disc^[5]^ was used as an interfacial layer between the LLZO and NMC-LPSCl cathode to improve contact. The cathode disk was uniaxially pressed at 160 MPa to densify the LPSCl catholyte. The full cell was then assembled by stacking the layers in the following sequence: Li/LLZO/PSPE/NMC-LPSCl. Electrochemical measurements were conducted at 75 °C under 1 MPa pressure in an Ar-filled glovebox, within a voltage range of 3 to 4.3 V *vs*. Li^+^/Li, using a BioLogic VMP-300 multichannel workstation.

**Calculations of energy density**

The achievable gravimetric and volumetric energy densities of Li/LLZO/NMC811-LPSCl full cells were calculated considering cathode with the areal capacity of 5 mAh cm^−2^, NMC811 active capacity of 200 mAh g^-1^, average cell voltage of 3.8 V, and total weight or volume of all cell components. The cell volume was calculated in the fully discharged state, which is the state that the battery would be assembled. The cell was assumed to consist of 1 layers of Al foil current collector with thickness of 16 µm on the cathode side and 1 layers of Cu foil current collector with thickness of 12 µm on the anode side. We assumed that the cathode is composed of 28 wt% of electrolyte (LPSCl), 2 wt% of carbon additives and 70 wt% NMC811, respectively. The thicknesses of NMC811 cathode, LLZO membrane and Li anode were 106.38 µm, 45 µm, and 10 µm, respectively. The densities of all cell components can be found in **Table S1**.


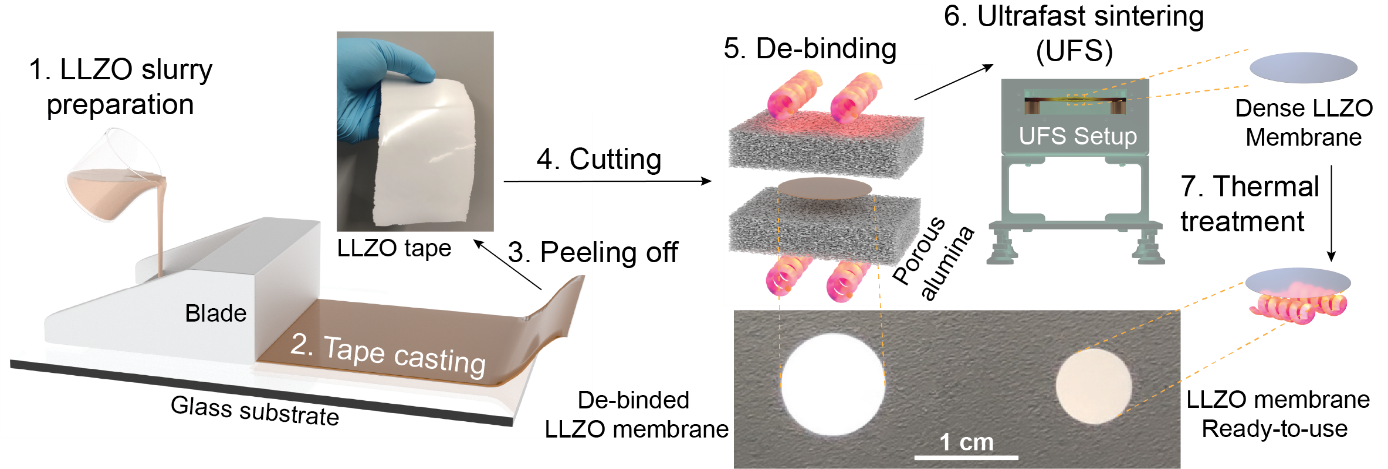


**Figure S1.** Schematic of the fabrication steps of dense LLZO membranes.

**
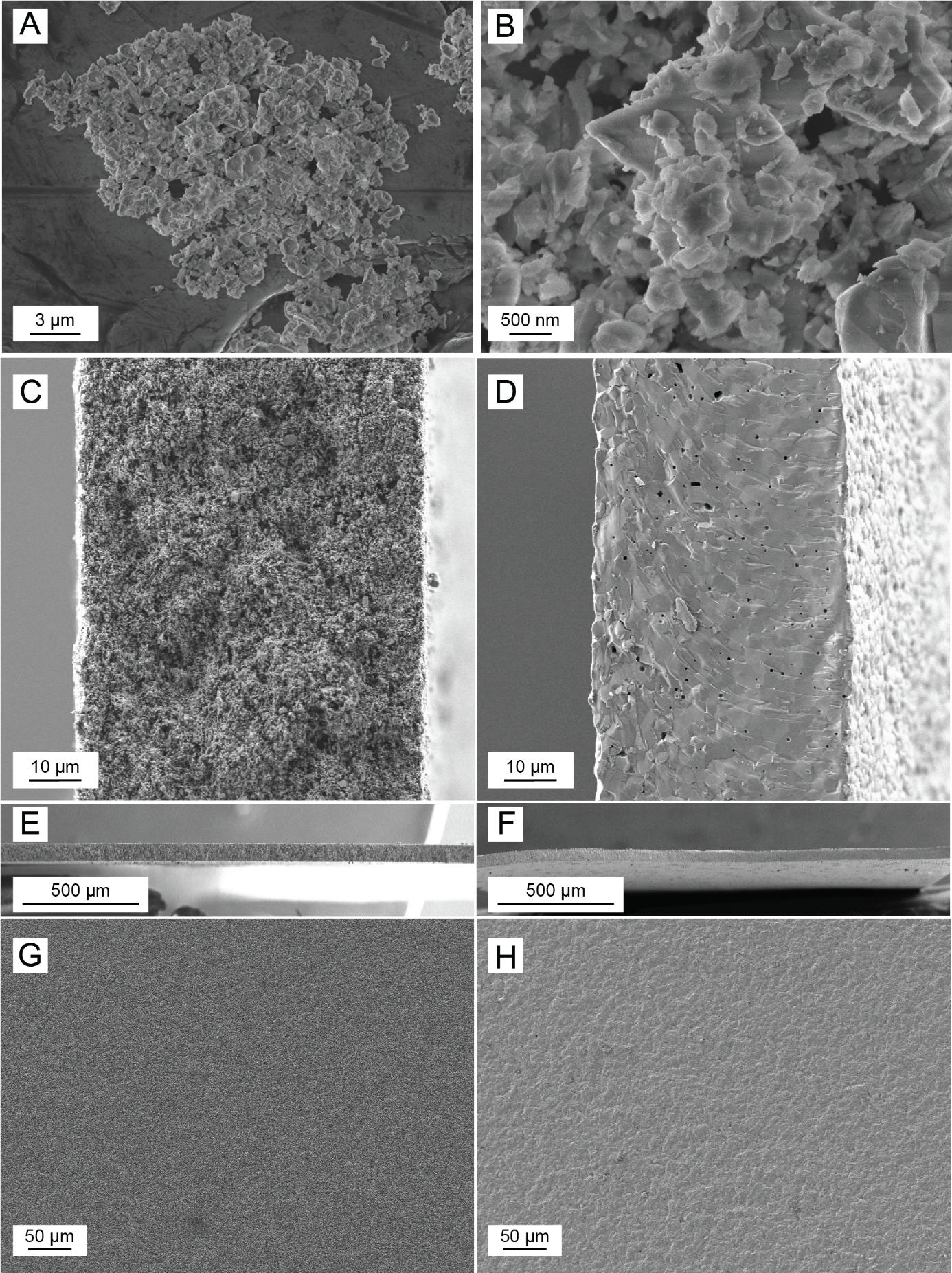
**

**Figure S2.** (a, b) SEM images of as-received LLZO powder. (c-f) Cross-sectional and (g, h) top-view SEM images of LLZO membranes after de-binding (c, e, g) and ultra-fast sintering (d, f, h).


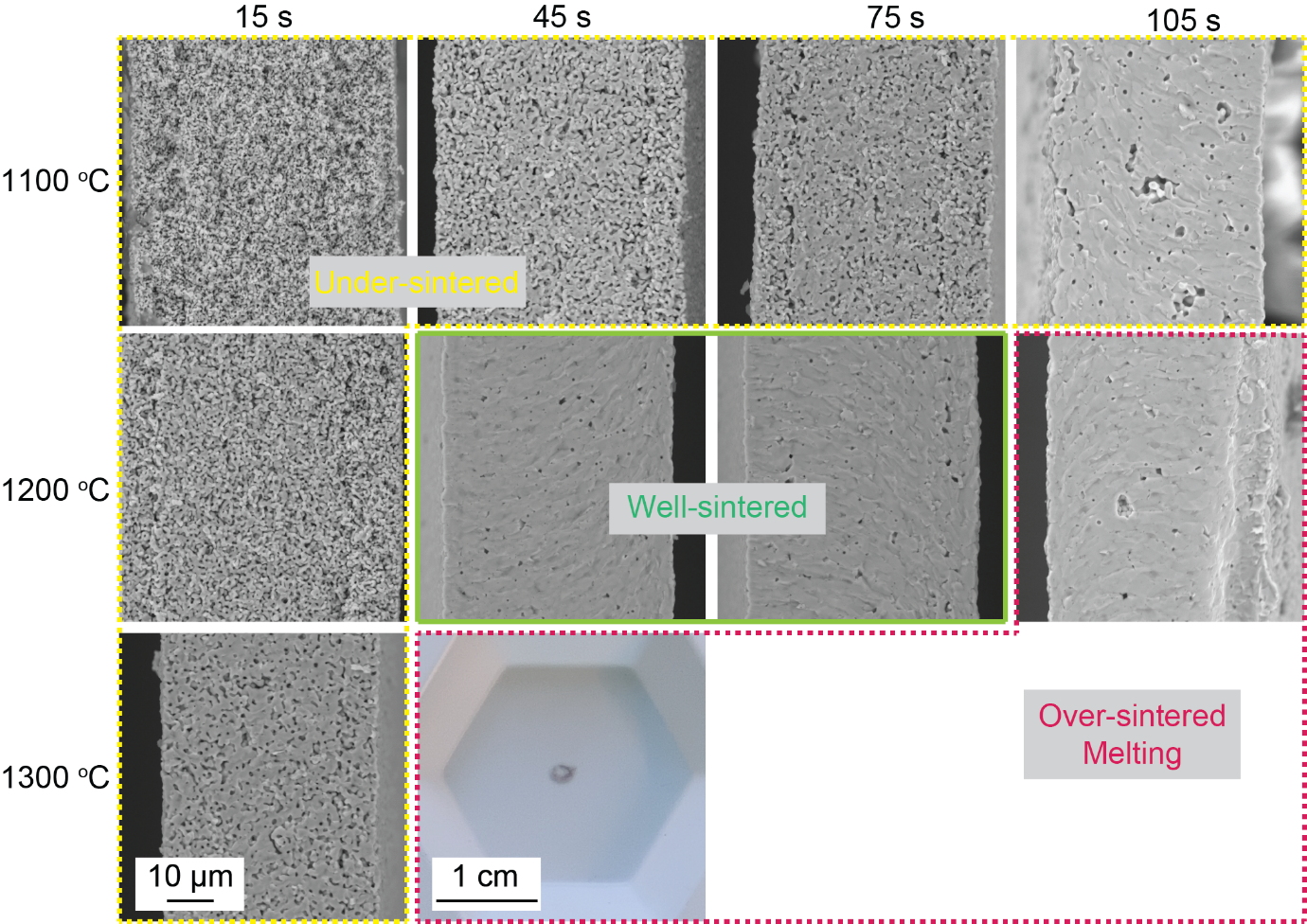


**Figure S3.** Cross-sectional SEM images of ultrafast-sintered LLZO membranes under different sintering conditions (time and temperature).


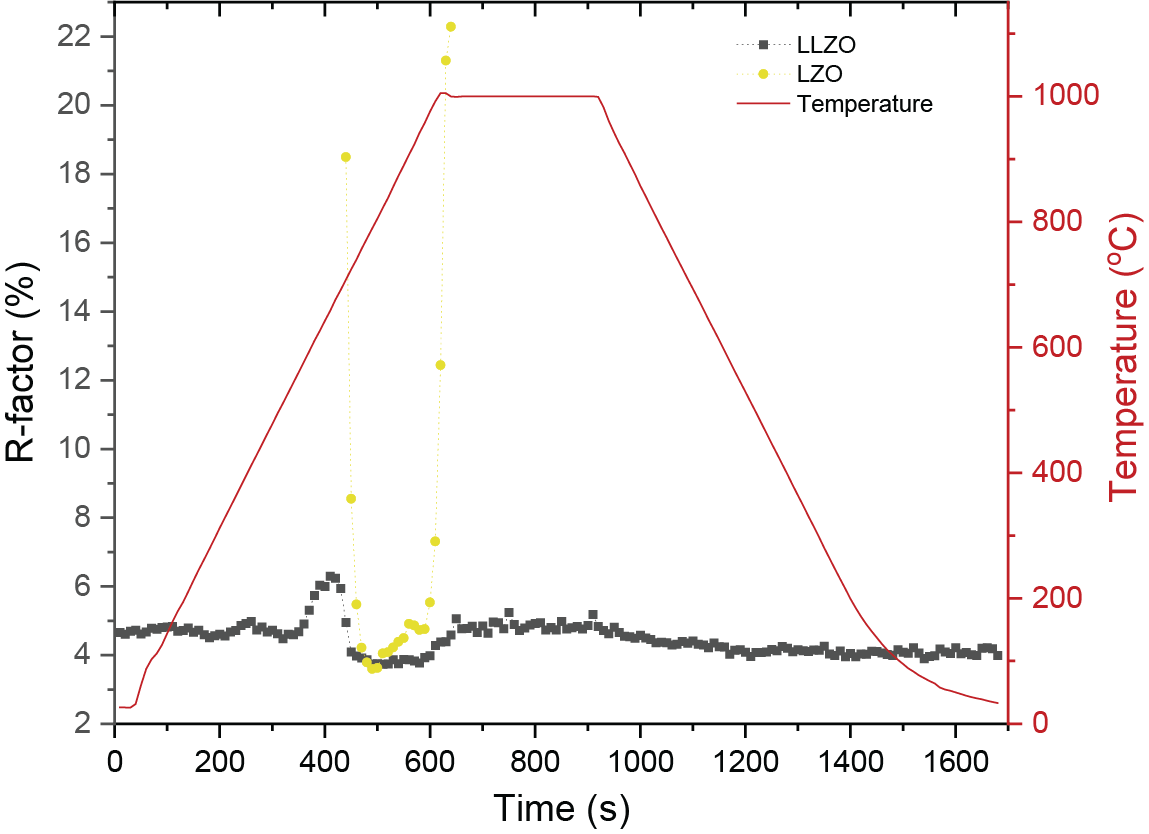


**Figure S4.** R-factor of the sequential Rietveld refinement shown in Figure 2a-c.


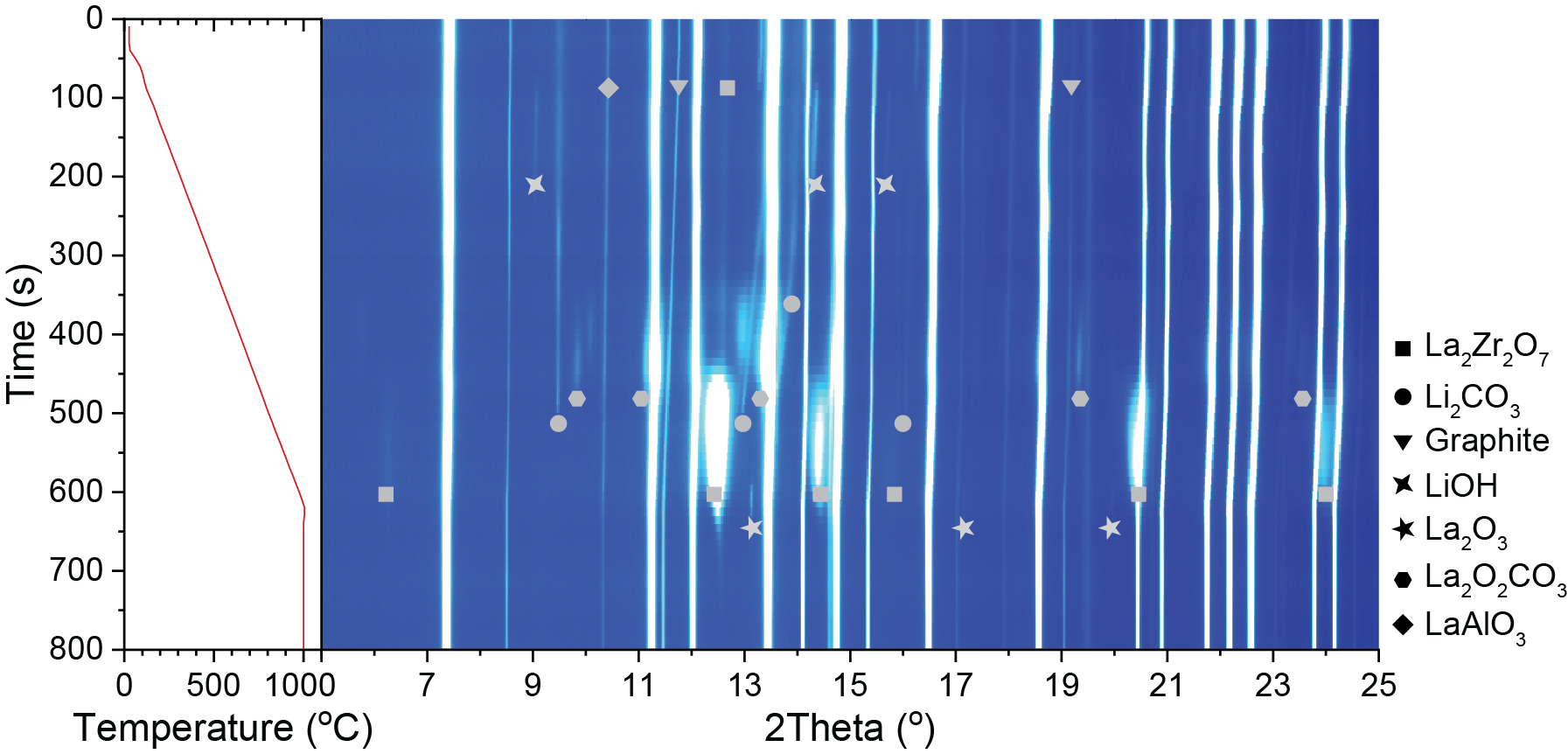


**Figure S5.** Enlarged SXRD map from Figure 2a with labeled peaks.


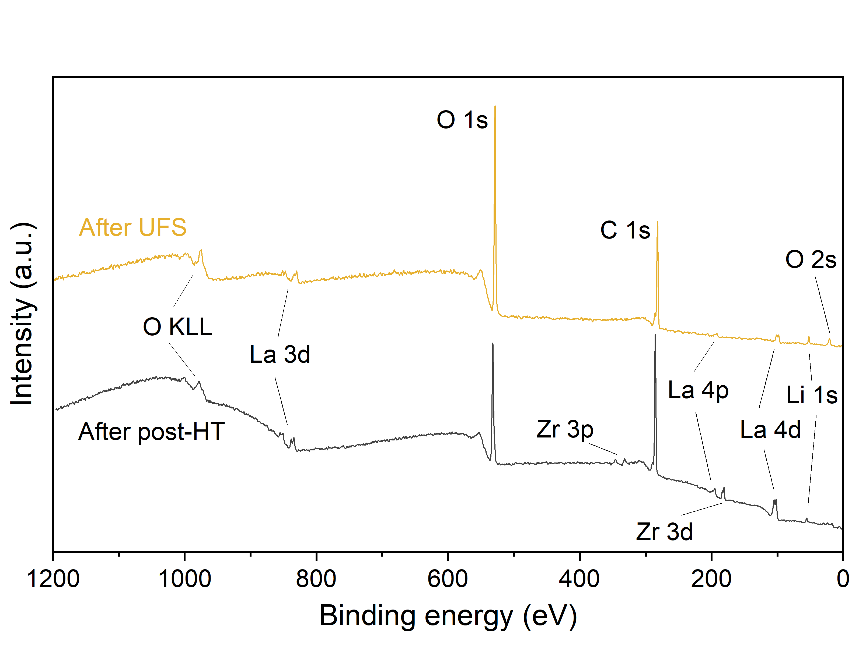


**Figure S6.** XPS surveys of LLZO membrane without (after UFS) and with (after post-HT) additional heat-treatment. Note: the LLZO membranes were transferred from the glovebox to XPS instrument without exposure to air.


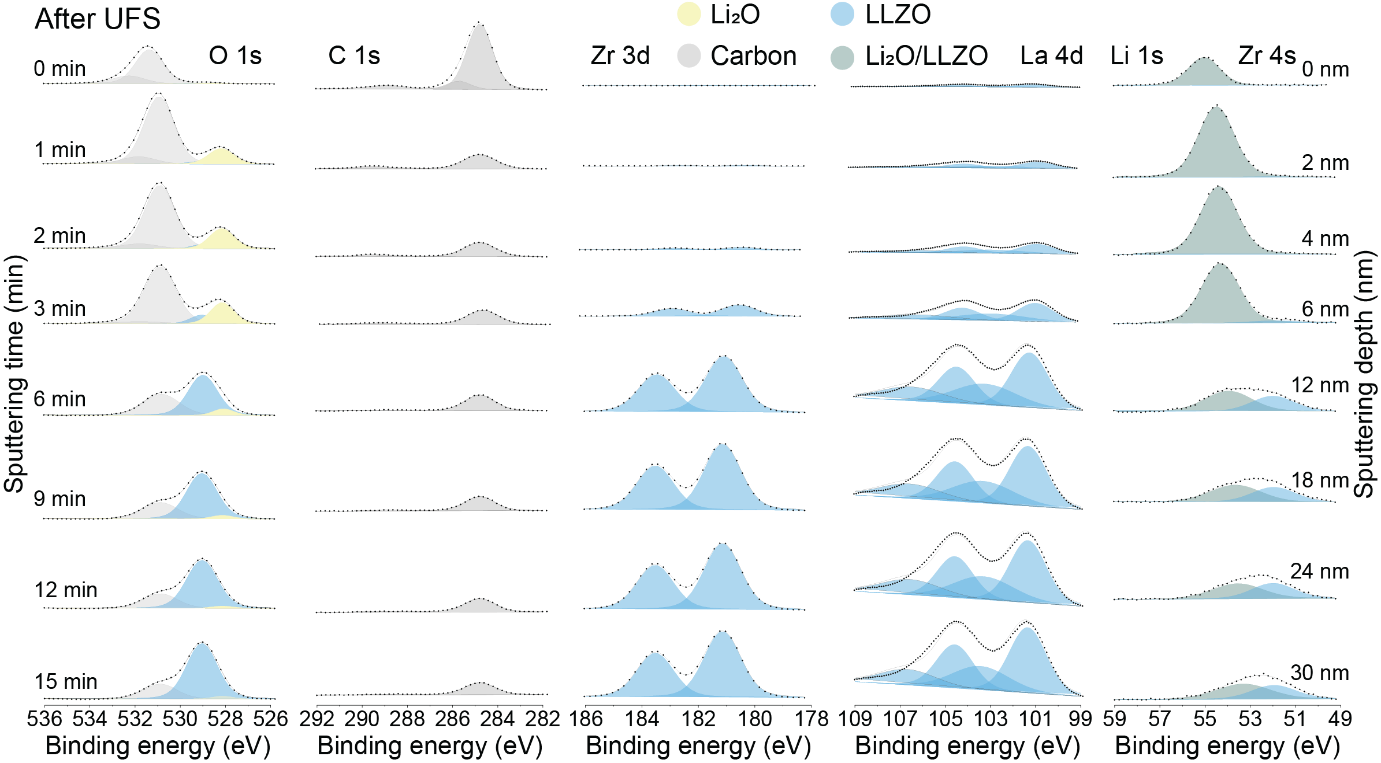


**Figure S7.** Charge-corrected O 1s, C 1s, Zr 3d, La 4d, Li 1s and Zr 4s XPS spectra measured on ultrafast-sintered LLZO membranes before and after sputtering for different times (1-15 min).


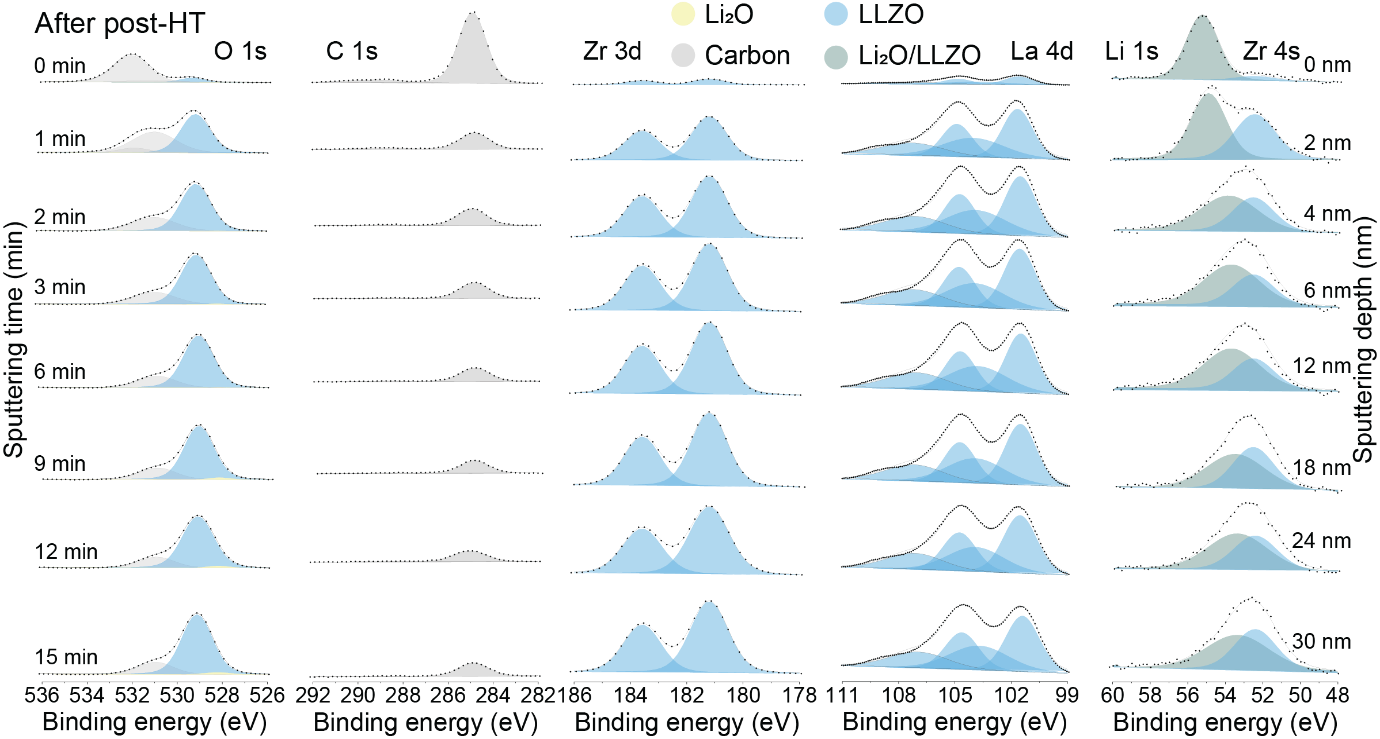


**Figure S8.** Charge-corrected O 1s, C 1s, Zr 3d, La 4d, Li 1s and Zr 4s XPS spectra measured on post-heat-treated ultrafast-sintered LLZO membranes before and after sputtering for different times (1-15 min).


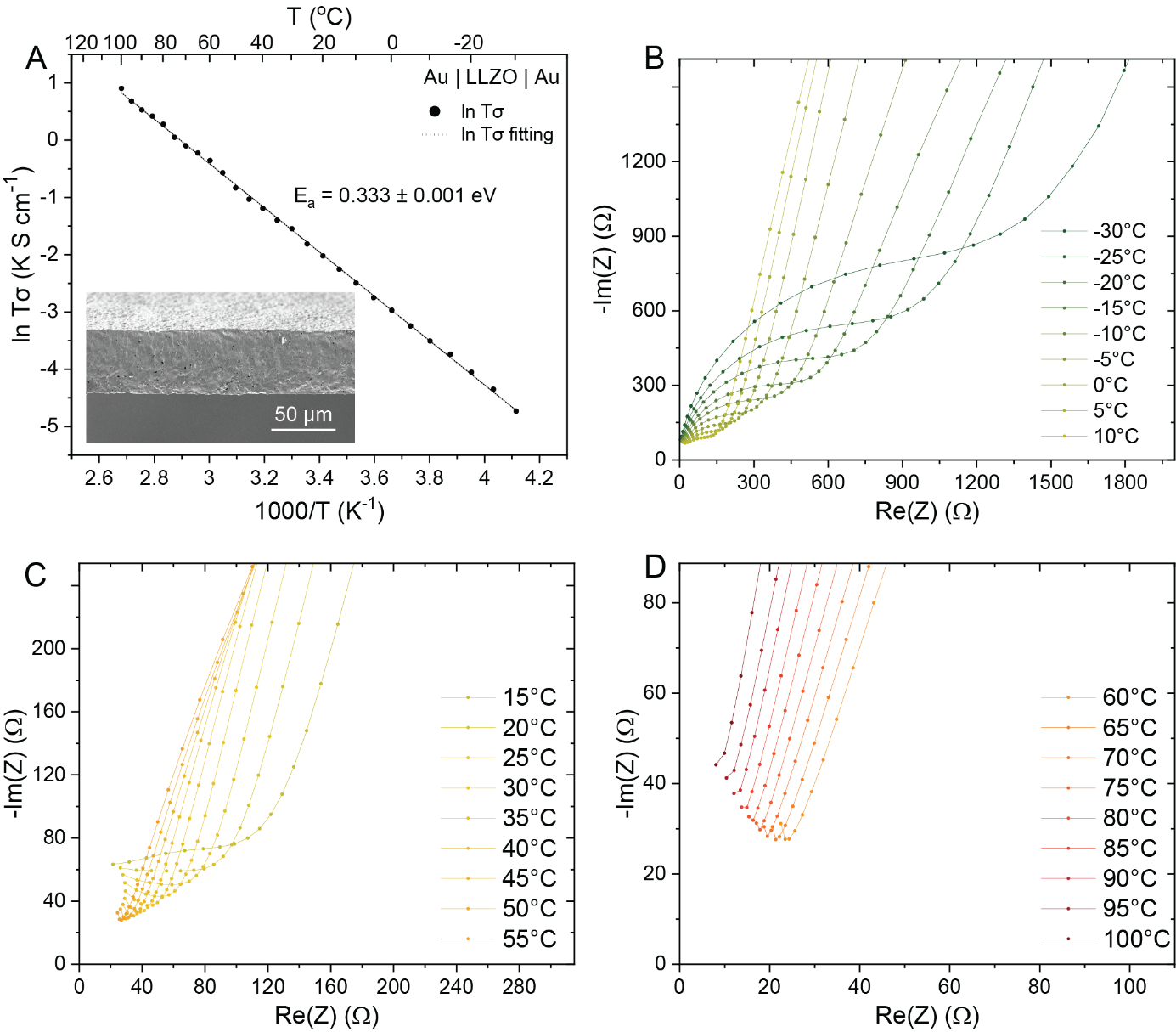


**Figure S9.** Arrhenius plot (a) and impedance spectra (b, c, d) of post-heat-treated ultrafast-sintered LLZO membranes measured using the Au/LLZO/Au symmetrical cell configuration, with a thickness of LLZO (45 µm) and the surface area of the Au electrodes (0.12566 cm^2^).


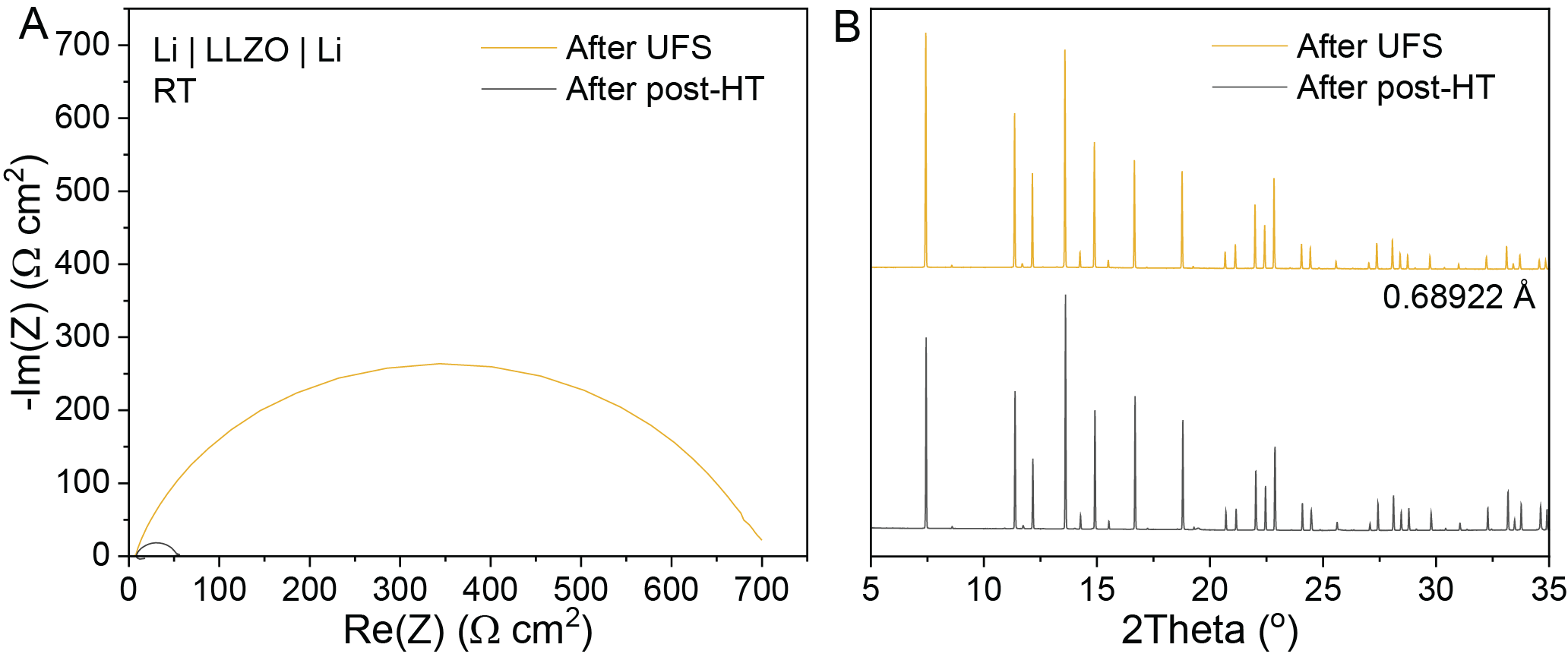


**Figure S10.** (a) Impedance spectra and (b) powder X-ray diffraction patterns of ultrafast-sintered LLZO membranes before and after additional post-heat-treatment steps. Electrochemical impedance spectroscopy measurements were performed at room temperature using the Au/LLZO/Au symmetrical cell configuration.


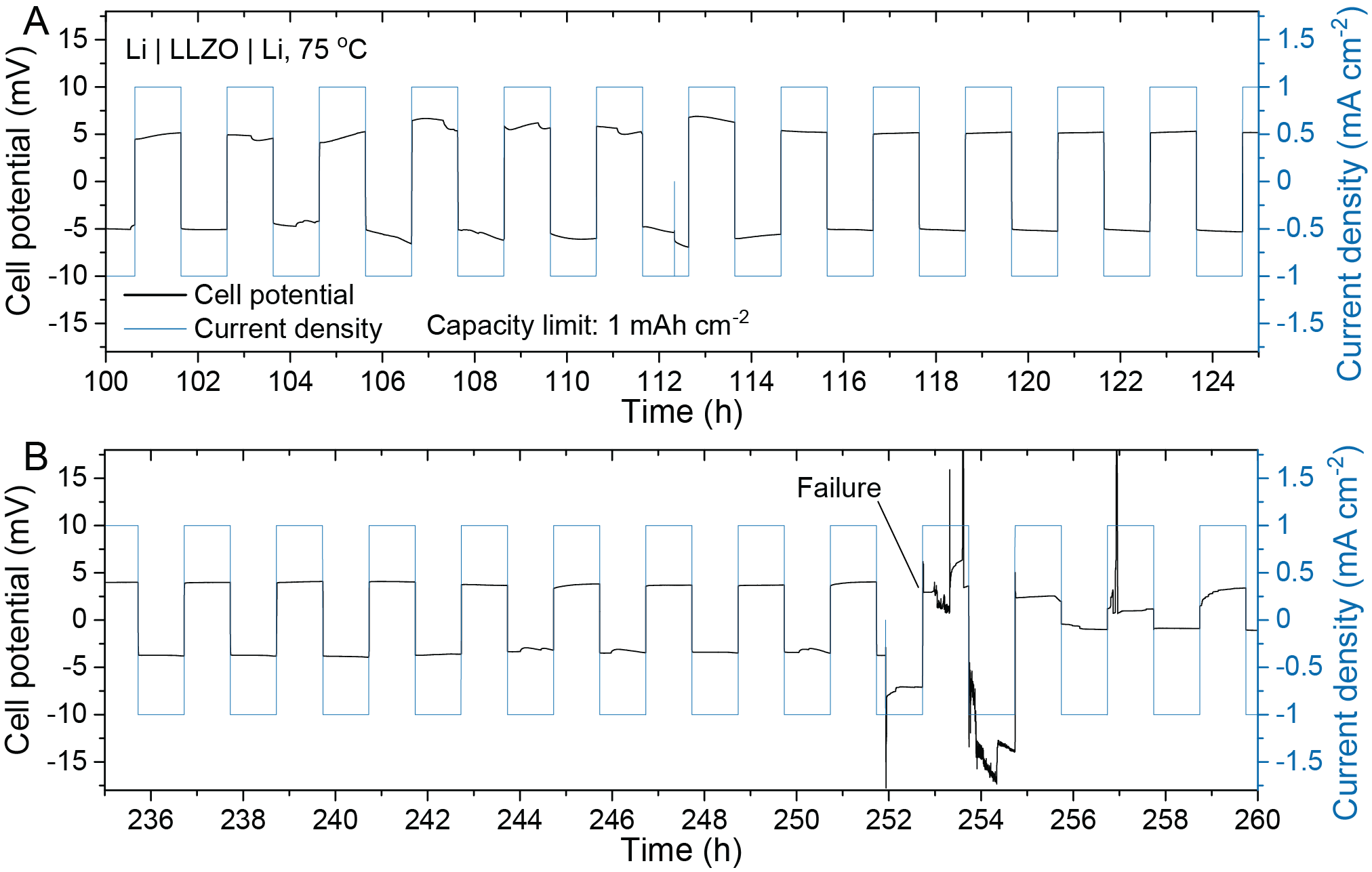


**Figure S11.** Enlarged voltage profile from 100 – 125 h (a) and 235 – 260 h (b) of the Li/LLZO/Li symmetrical cell in Figure 4b. The disturbance of overpotential was caused by temperature changes and short pauses due to experimental maintenance of the sealed vacuum furnace integrated within an argon-filled glovebox rather than a soft short-circuit. The failure of the symmetrical cell happened at around 253 h with a clear short-circuit, defined by a sudden drop in the voltage.


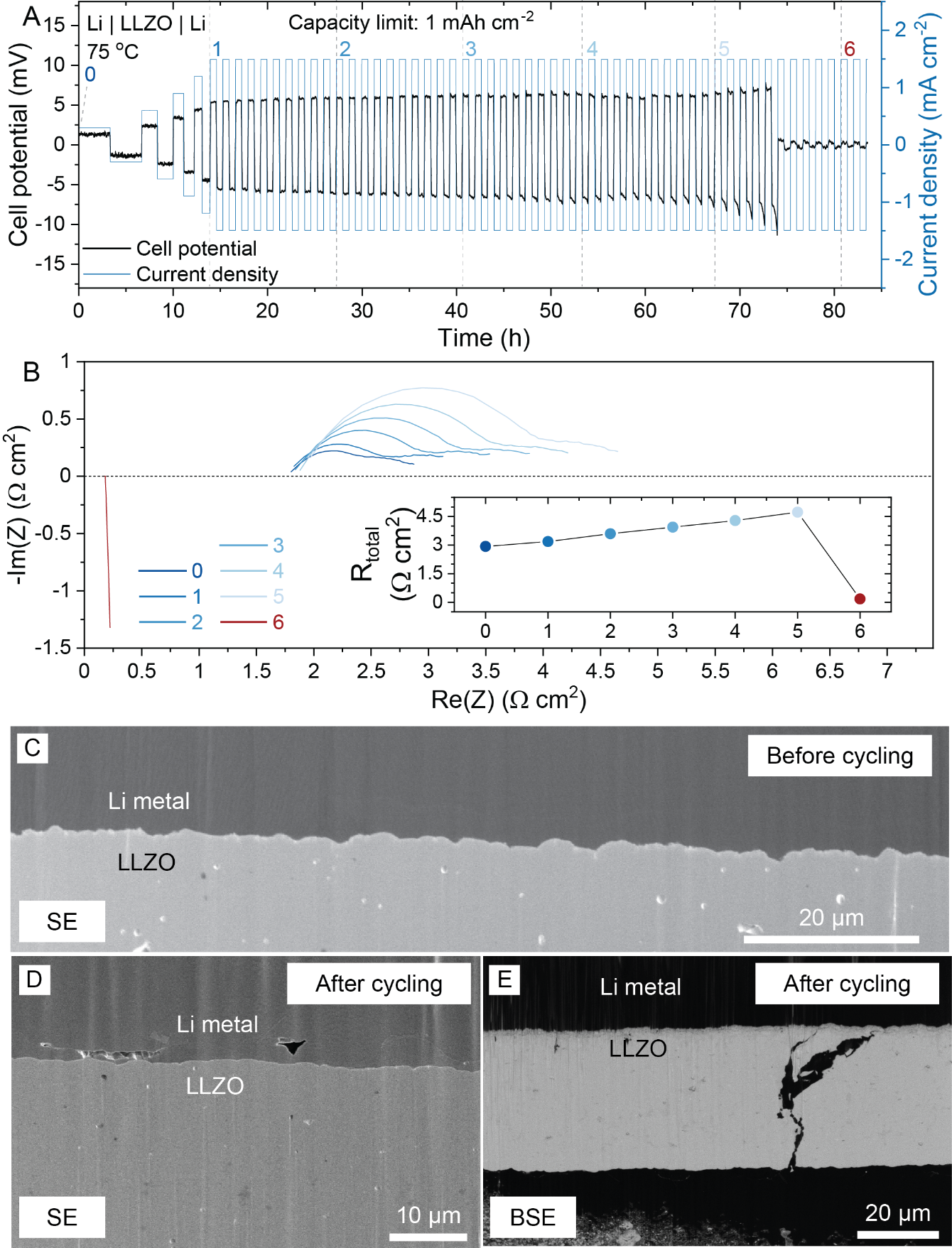


**Figure S12.** (a) Voltage profile of a Li/LLZO/Li symmetrical cell measured at a current density of 1.5 mA cm^-2^ with an areal capacity limitation of 1 mAh cm^-2^ per half cycle. (b) Impedance spectra of the Li/LLZO/Li symmetrical cell after every 10^th^ cycle. The change of total resistances as a function of EIS cycle number refined from the fitting of corresponding impedance spectra is shown in the inset. FIB-SEM image before (c) and after (d-e) cycling of a Li/LLZO/Li symmetrical cell. The post-mortem SEM analysis using secondary electrons (SE) and back-scattered electrons (BSE) revealed void formation and the presence of Li dendrites, respectively. These findings correlate with the observed changes in total resistance during cycling.


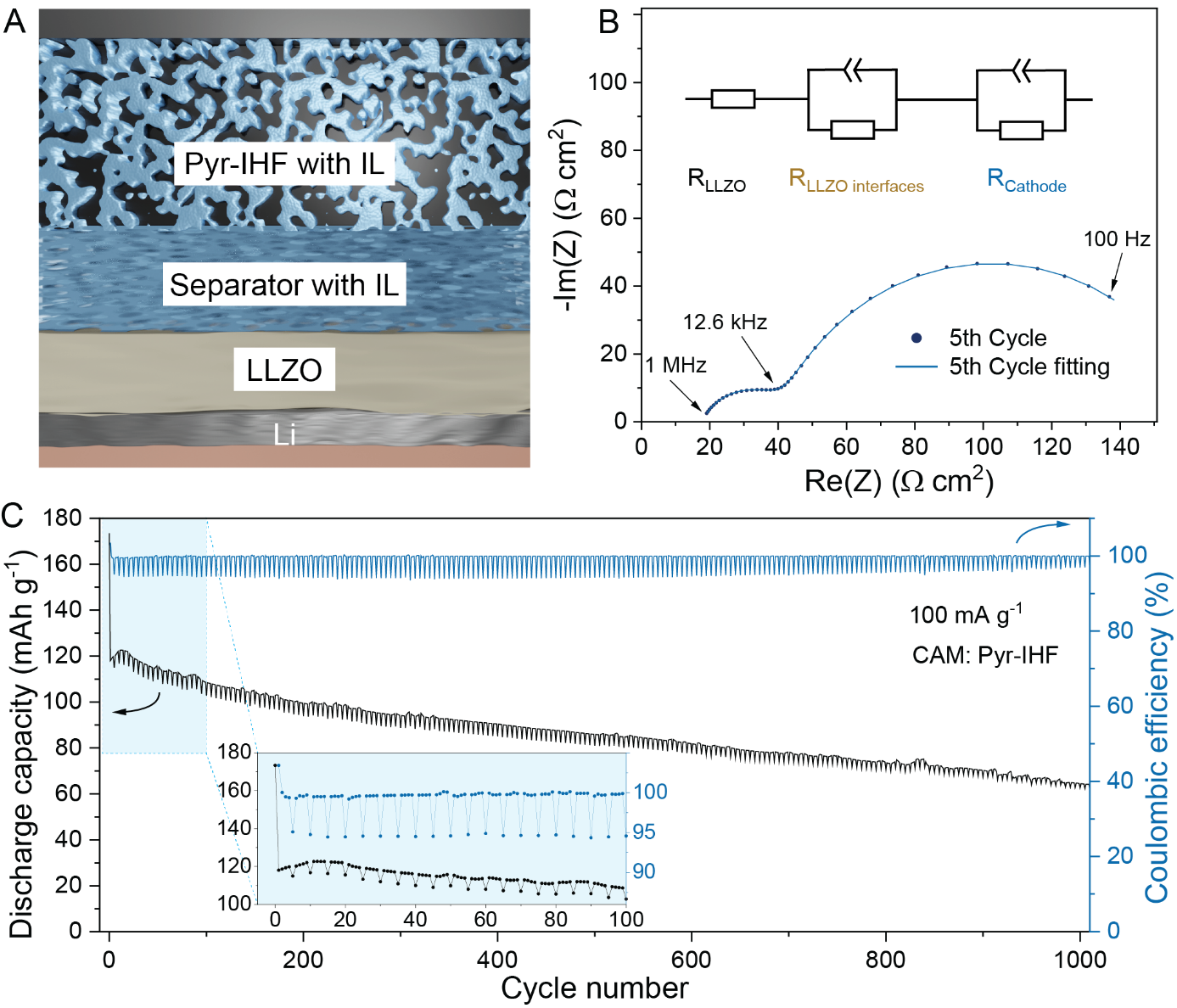


**Figure S13.** (a) Schematic configuration of the Li/LLZO/Pyr-IHF full cell. (b) Impedance spectra of Li/LLZO/Pyr-IHF full cell measured in charge state after 5th cycle (inset: equivalent circuit used to fit impedance spectra). (c) Capacity stability and Coulombic efficiency of the Pyr-IHF cathode measured at a current density of 100 mA g^-1^, corresponding to an areal current density of 0.041 mA cm^-2^ (inset: capacity stability and Coulombic efficiency of the Pyr-IHF cathode for the first 100 cycles). Note: repeatable capacity drops over cycling are caused by electrochemical impedance spectroscopy measurements during which the cell was discharged by ca. 5 mAh g^-1^.


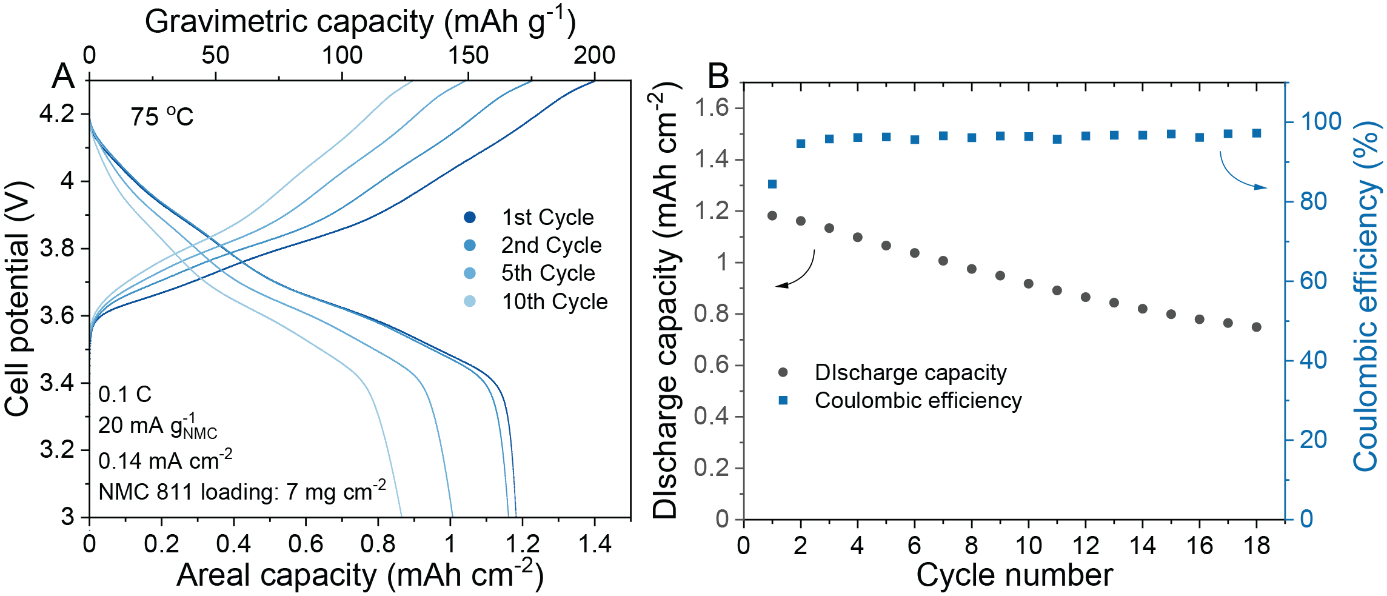


**Figure S14.** Electrochemical performance of a Li/LLZO/NMC811-LPSCl all-solid-state battery. The mass loading of NMC811 was ca. 7 mg cm^-2^. (a) Galvanostatic charge-discharge voltage profiles, capacity retention (b) and coulombic efficiency (b) of the Li/LLZO/NMC811-LPSCl all solid-state battery measured at a current density of 20 mA g^−1^ between 3 V and 4.3 V vs. Li^+^/Li.

**Table S1.** Parameters used in calculating energy density of Li/LLZO/NMC811-LPSCl full cell.

| **Parameters** | **Unit** | **Value** |
| --- | --- | --- |
| Thickness of Al current collector | µm | 16 |
| Density of NMC811 | g cm^-3^ | 4.77 |
| Loading of NMC811 | mg cm^-2^ | 25 |
| Density of Carbon additives | g cm^-3^ | 1.8 |
| Density of LPSCl | g cm^-3^ | 2.0 |
| Ratio of NMC-Carbon-LPSCl | - | 70:2:28 in weight |
| Theoretical capacity of NMC811 | mAh g^-1^ | 200 |
| Average voltage | V | 3.8 |
| Thickness of cathode in total | µm | 106.38 |
| Density of LLZO | g cm^-3^ | 5.1 |
| Thickness of LLZO membrane | µm | 45 |
| Thickness of Li anode | µm | 10 |
| Thickness of Cu current collector | µm | 12 |
| **Gravimetric energy density** | **Wh kg^-1^** | **258** |
| **Volumetric energy density** | **Wh L^-1^** | **888** |

**References**

[1] F. Okur, H. Y. Zhang, D. T. Karabay, K. Muench, A. Parrilli, A. Neels, W. Dachraoui, M. D. Rossell, C. Cancellieri, L. P. H. Jeurgens, K. V. Kravchyk, M. V. Kovalenko, *Adv Energy Mater* **2023**, *13* (15).

[2] J. F. Baumgärtner, M. Wörle, C. P. Guntlin, F. Krumeich, S. Siegrist, V. Vogt, D. C. Stoian, D. Chernyshov, W. van Beek, K. V. Kravchyk, *Adv. Mater.* **2023**, *35* (49), 2304158.

[2] K. P. Marshall, H. Emerich, C. J. McMonagle, C. A. Fuller, V. Dyadkin, D. Chernyshov, W. v. Beek, *J. Synchrotron Radiat.* **2023**, *30* (1), 267.

[3] D. Chernyshov, V. Dyadkin, W. van Beek, A. Urakawa, *Acta Crystallogr. A Found. Adv.* **2016**, *72* (4), 500.

[5] F. Okur, Y. Sheima, C. Zimmerli, H. Zhang, P. Helbling, A. Fäh, I. Mihail, J. Tschudin, D. M. Opris, M. V. Kovalenko, K. V. Kravchyk, *Chemsuschem* **2024**, *17* (3).
